# Modelling and mapping tick dynamics using volunteered observations

**DOI:** 10.1186/s12942-017-0114-8

**Published:** 2017-11-14

**Authors:** Irene Garcia-Martí, Raúl Zurita-Milla, Arnold J. H. van Vliet, Willem Takken

**Affiliations:** 1 Department of Geo-Information Processing (GIP), Faculty of Geo-Information and Earth Observation (ITC), University of Twente, Enschede, The Netherlands; 2 Department of Environmental Sciences, Wageningen University, Wageningen, The Netherlands; 3 Department of Plant Sciences, Wageningen University, Wageningen, The Netherlands

**Keywords:** Tick dynamics, Random forest, Volunteered geographic information (VGI), Data analysis, Environmental modelling

## Abstract

**Background:**

Tick populations and tick-borne infections have steadily increased since the mid-1990s posing an ever-increasing risk to public health. Yet, modelling tick dynamics remains challenging because of the lack of data and knowledge on this complex phenomenon. Here we present an approach to model and map tick dynamics using volunteered data. This approach is illustrated with 9 years of data collected by a group of trained volunteers who sampled active questing ticks (AQT) on a monthly basis and for 15 locations in the Netherlands. We aimed at finding the main environmental drivers of AQT at multiple time-scales, and to devise daily AQT maps at the national level for 2014.

**Method:**

Tick dynamics is a complex ecological problem driven by biotic (e.g. pathogens, wildlife, humans) and abiotic (e.g. weather, landscape) factors. We enriched the volunteered AQT collection with six types of weather variables (aggregated at 11 temporal scales), three types of satellite-derived vegetation indices, land cover, and mast years. Then, we applied a feature engineering process to derive a set of 101 features to characterize the conditions that yielded a particular count of AQT on a date and location. To devise models predicting the AQT, we use a time-aware Random Forest regression method, which is suitable to find non-linear relationships in complex ecological problems, and provides an estimation of the most important features to predict the AQT.

**Results:**

We trained a model capable of fitting AQT with reduced statistical metrics. The multi-temporal study on the feature importance indicates that variables linked to water levels in the atmosphere (i.e. evapotranspiration, relative humidity) consistently showed a higher explanatory power than previous works using temperature. As a product of this study, we are able of mapping daily tick dynamics at the national level.

**Conclusions:**

This study paves the way towards the design of new applications in the fields of environmental research, nature management, and public health. It also illustrates how Citizen Science initiatives produce geospatial data collections that can support scientific analysis, thus enabling the monitoring of complex environmental phenomena.

## Background

Tick populations and tick-borne infections like Lyme borreliosis have steadily increased since the mid-1990s. This concurrent increase has been observed in various European countries [19, 23], in the US [48] and in Canada [31]. In the Netherlands, periodic national studies among general practitioners (GPs), revealed a consistent two-decade rising trend in the number of tick bites consultations and Lyme borreliosis diagnoses [22], that only showed a first sign of stabilization recently. Still, more than 20,000 people per year develop Lyme borreliosis in the Netherlands and its disease burden is substantial, especially in patients who develop chronical symptoms [21].

Scientists of different fields have investigated this global increase of tick populations and tick-borne infections, converging upon two main causes: global environmental changes are altering the spatio-temporal dynamics of ticks [29, 47] and socio-economic changes are changing the spatial patterns of human populations around urbanized areas, increasing the human exposure to ticks [38, 40, 53]. Tick dynamics are complex ecological processes driven by numerous factors (i.e. wildlife, weather, vegetation, landscape). Understanding the interactions between these factors and tick dynamics is crucial to develop models capable of forecasting the incidence and distribution of ticks and tick-borne diseases [12, 33].

Models predicting the spatio-temporal distribution of ticks are needed to implement control measures which mitigate future disease infections [9, 18] or help managing public health risks [29]. However, the development of such models is not straightforward due to several issues. First, it is unclear what the best set of environmental predictors are. Past studies have found correlations between different combinations of biotic and abiotic factors and tick dynamics, but the spatio-temporal scale of these experiments is diverse enough to pose difficulties in drawing general conclusions. For instance, Berger et al. [2, 3] found a link between relative humidity and the seasonal abundance of ticks at the regional level. Dantas-Torres and Otranto [10] found weak correlations at local scale between monthly temperature, evapotranspiration and saturation deficit with tick abundances, whereas [42] found links (in laboratory conditions) between the saturation deficit and the number of questing ticks. Second, it is often unclear at what time scales the different predictors operate. Previous studies have found linear correlations between tick abundances and environmental predictors at multiple temporal scales [2, 3, 50]. However, the temporal sparsity of the tick sampling or the use of short-term time series question if these correlations are scalable to long-term time series at the country level. Third, tick dynamics are complex phenomena that traditionally have been modelled with linear methods. Two of the well-known disadvantages of classical linear methods is that they are not capable of finding non-linear interactions between variables (except when explicitly included a priori), and do not properly handle large numbers of predictors (e.g. due to collinearity). However, such data are a reality when modelling complex natural phenomena.

In this work, we address the above-mentioned issues by modelling nine years of monthly data on Active Questing Ticks (AQT) collected by volunteers on 15 different locations in the Netherlands. This modelling exercise includes a wide array of (a) biotic predictors and, by applying an ensemble regression method (i.e. random forest), we aim at identifying the most important variables to model AQT at multiple time-scales. Building such AQT dynamic model allows us to explore and map tick’s seasonality across the Netherlands. We envision applications of this model in the fields of environmental and ecological research, nature management and public health, which hopefully will reduce the incidence of Lyme disease.

## Ticks and environment

### Tick sampling

Ticks are blood sucking arthropods capable of transmitting a wide variety of pathogens (e.g. bacteria, viruses) which cause disease in humans [19]. Deciduous or mixed forests in temperate and humid regions, which are inhabited by different mammalian species (e.g. deer, rodents), create optimal habitats sustaining ticks life cycle [33]. Ticks quest at the top of vegetation or litter layer, waiting for a human or animal host to attach and feed. This behavior is used to determine tick populations in a particular location. To do so, two manual monitoring techniques are used: flagging and dragging. Flagging consists on sweeping a squared cloth attached to a pole on one side upon the litter or vegetation layers, whereas dragging consists in attaching the previous material to a rope, which the investigator can pull along the study area [45]. In both cases, ticks that are touched by the cloth attach to it, allowing researchers to count the number of ticks in its different life stages (i.e. larvae, nymph, or adult). Both techniques have been widely used in small scale biological studies to acquire raw data on tick counts that can be later incorporated in a scientific workflow [10, 11, 13, 15, 41].

### Environmental factors

Ticks are particularly susceptible to environmental conditions because of their high surface-to-volume ratio, which makes them experience water losses through their exoskeleton, and their lack of thermal inertia, which makes them vulnerable to extreme weather conditions [33]. The following sub sections list the environmental variables used in our work and sketch their impact on tick dynamics.

#### Weather data

Temperature determines the start of the questing season, tick population development rate and the chances of survival through the winter season [32, 40, 52]. Precipitation and relative humidity are crucial to sustain tick populations in nature. Precipitation is necessary during the summer season [25], but extreme precipitation events (i.e. drought and heavy rain) may prevent the development of new tick populations [33]. Long-lasting and adverse humidity conditions have been linked to an increased mortality among nymphal ticks and this, in turn, may decrease the total number of cases of Lyme disease [2, 3]. Some studies suggest that nymphal ticks can desiccate within 48 h if the humidity conditions at ground level are suboptimal [2, 3]. Additionally, relative humidity and temperature can be used to calculate the saturation deficit and vapor pressure. Saturation deficit has been used in a previous and thorough study to understand the role of humidity in tick survival [42] and vapor pressure has been identified as a major indicator of tick habitat suitability [8]. In some studies, evapotranspiration has been used as a proxy for vapor pressure deficit [44].

Weather datasets are publicly available at the online data center of the Royal Netherlands Meteorological Institute (KNMI).^1^ We downloaded daily gridded layers of temperature, precipitation, evapotranspiration and relative humidity for the period 2005–2014. From temperature and relative humidity, we obtained saturation deficit and vapour pressure [30, 42]. The temporal resolution of the weather datasets and the tick sampling is different, since the former are available at daily temporal resolution, whereas the latter is carried out on a unique day each month. To match both resolutions, it is necessary to aggregate the weather variables to a coarser temporal scale in a way that reflect the impact later caused on the tick count.

#### Vegetation data from satellites

Ticks are sensitive to local environmental conditions, such as the thickness of forest canopy or soil moisture at the ground level [29]. Earth observation satellites allow the monitoring of these environmental conditions over large areas. In this work, we used three vegetation indices to characterize local environmental conditions: the Normalized Difference Vegetation Index (NDVI), the Enhanced Vegetation Index (EVI) and the Normalized Difference Water Index (NDWI). Previous studies have demonstrated that fluctuations in NDVI, which has traditionally been used to measure the greenness and the density of vegetation, correlate well with fluctuations in the number of nymphs and adult ticks and that NDVI can be used as a proxy to find suitable tick habitats [11, 39]. More recent studies show that novel vegetation indices like EVI or NDWI are better estimators of tick populations [1] and Lyme disease incidence [37].

Vegetation indices are publicly available in the Google Earth Engine (GEE) platform.^2^,^3^ GEE is a free image processing cloud platform for environmental analysis, which aggregates and integrates products coming from different Earth observation sensors, such as the Moderate-Resolution Imaging Spectroradiometer (MODIS). MODIS provides daily global imagery at 250, 500 and 1000 m of spatial resolution. However, due to the persistent cloud coverage over the Netherlands we used MODIS composite products. In particular, we used the MCD43A4 product, which provides the NDVI, EVI and NDWI indices derived from the daily surface reflectance at a pixel size of 500 m, using data of the previous 16 days. It is important to note that this product is released every 8 days, so there is a 50% of temporal overlap between each composite, meaning that the vegetation signal will contain smooth changes.

#### Land cover, tick habitat and mast years

Land cover is another important factor in the field of tick ecology because it influences tick survival and determines the chances of human-tick contact. Ticks prefer habitats where the vegetation prevents reaching desiccation conditions and where hosts (e.g. deer, rodents, mice) species are present. Complex landscapes, in which multiple land covers are intertwined in a small area unit, increase the probability of contact between ticks and their human or animal hosts [17, 26, 27, 51]. For land cover we use the 7th release of the national land cover database or LGN (Landelijk Grondgebruik Nederland^4^). This database was produced in 2012 and contains information for 39 classes at 25 m.

The sampling sites are located in forested areas with specific types of vegetation (i.e. deciduous and coniferous forest, grasses, and bushlands). The plant associations in these sites contribute determining the presence of wildlife species in each location, by providing forage or shelter, and subsequently, tick populations move with them. Previous studies have demonstrated that deciduous forests present higher abundances of AQT than coniferous forest, and also that a dense shrub layer has a positive effect on tick populations [49]. Gassner et al. [15] gives a thorough description of the plant associations and habitat characteristics found in the surroundings of each transect of the flagging sites.

Mast seeding is a natural phenomenon in which certain plant species synchronously produce an abnormal amount of acorns and nuts [46]. This overproduction feeds a wide range of animal species and contributes to a steep increase of their populations for the next season. When the populations of rodents, deer and other tick host species increase, the same occurs with tick populations [34, 36]. Dutch volunteers from the Mammal Association (http://www.zoogdiervereniging.nl) quantify each year the amount of acorns produced for beech, oak and American oak. The amount of acorns is classified in a categorical scale that goes from 0 to 5 depending on how strong the mast year.

## Data

This work relies on a unique dataset of tick dynamics collected by volunteers in the context of a project of participatory modelling. This dataset was enriched with a set of environmental variables extracted for each sampling location. For this, we collected and preprocessed weather and satellite data, and included biological data regarding habitat and mast years. The remaining of this section first contains a description of the volunteered tick counts data (“Volunteered tick counts reports” section), and then we explain the process of feature engineering carried out to create a series of predictors that characterize tick dynamics as monitored by volunteers.

### Volunteered tick counts reports

**Fig. 1:**
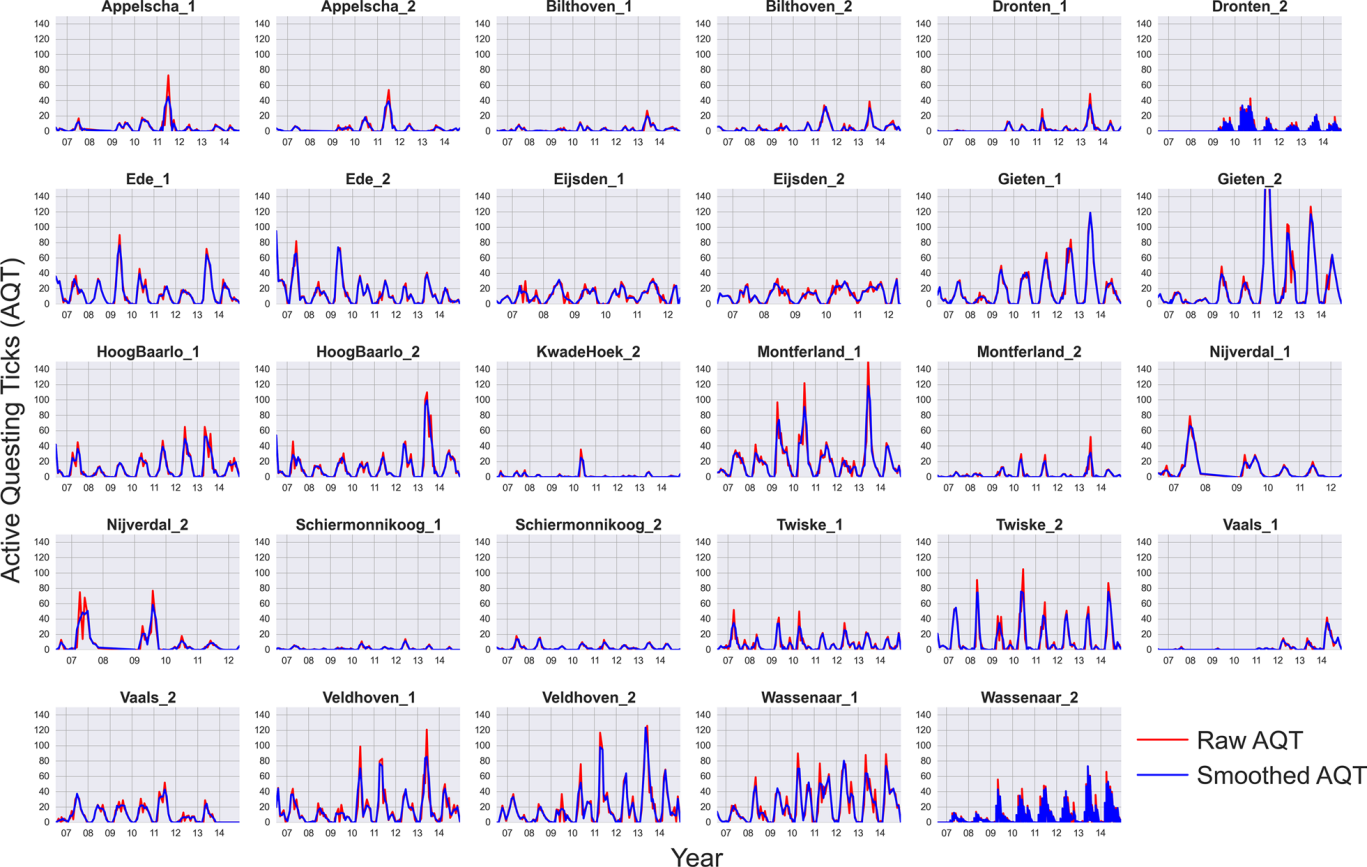
Monthly time-series (2006–2014) of active questing ticks (AQT) per transect. Each subplot shows both the number of ticks counted by the volunteer (red) and the Savizky-Golay smoothed version of this signal (blue)

In the context of the Dutch phenological network Nature’s Calendar (www.natuurkalender.nl) every month since July 2006, a group of volunteers sampled AQT on 24 forest sites. This joint effort aimed to quantify and understand the spatial and temporal dynamics of ticks and the Borrelia bacteria that can cause Lyme disease [15]. Out of the 24 sites participating in the research project, we were able to include data from 15 sites, which represent a total of 3073 observations collected by volunteers. We excluded the sites in which the sampling stopped in an early stage of the project, or the site was sparsely sampled in time. At each site, volunteers sampled two transects, separated from each other several hundred meters. Ticks were collected using a technique called “dragging”, in which the volunteer drags a 1 m^2^ cloth over the low vegetation of each transect for 100 m, turning the cloth every 25 m to count the number of larvae, nymphs and adult ticks. This study focuses on the nymphs because they pose the highest risk for humans to get a tick bite. Figure 1 shows the raw number of nymphs per transect and per month. The number of AQT across all sites present strong spatial and temporal variations: (1) some transects present a more continuous and recurrent shape, whereas others have an erratic tick count (e.g. Gieten vs. Bilthoven); (2) some transects produce very different yields, from low tick counts to high peaks (e.g. Veldhoven vs. Eijsden); (3) transects within a sampling site may yield a different number of ticks, even though they are close in space and sampled on the same day (e.g. Montferland). The reasons of these strong local and seasonal variations are still poorly understood, but previous works have found clear links between tick populations and the abundance of small mammals in the area [35], mast years [24] or warming weather conditions [25, 48], which are major influences over tick dynamics, as seen in “Environmental factors” section.

Volunteered projects have proved useful to acquire information at a timely and fine spatial scale, but the quality and the amount of uncertainty of such data collections is difficult to measure [4, 5, 16]. A visual inspection of Fig. 1 shows that the monthly tick counts signal presents an irregular and noisy shape. A closer descriptive analysis of the raw data reveals that out of 3073 records in the dataset, around one-third of the samples are zeros, and a small proportion of samples present high peaks. Zero AQT means that a volunteer visited a site for tick sampling on a particular date and no ticks were caught questing, whereas a peaky AQT means the ticks were very active on that day.

To assess the potential impact that zero and peaky AQT may have in our modelling process, we created four versions of the original dataset, which vary in the amount of zeroes and peaky observations. In two datasets we removed all samples with a zero AQT within the tick season (i.e. 1st March until 31st October) and half of the samples with a zero AQT outside the season. This creates a group with two datasets with a reduced amount of zeros, and a second group with two datasets which are not modified with respect to the original. After this step, we applied a smoothing process to only one of the datasets of each group. We chose a Savitzky–Golay filter to mitigate the effect of peaky AQT in the modelling process, whereas the other dataset was kept with the original AQT signal. In this way, the modelling process accounts for the possible effect of extreme observations to fit the AQT signal, and helps distinguishing whether varying levels of noise is hampering the learning process of the chosen modelling algorithm.

### Characterizing the environment

Feature engineering is a common process in the machine learning field to obtain new predictors from original data sources, which incorporate the knowledge of a domain to create predictive models. In our case, we obtained a set of features, based in the theoretical grounds described in “Tick sampling” and “Environmental factors” sections, which aim to that aim to characterize the environmental conditions in each tick sampling site. Thus, this work uses 101 features (Table 1) classified in five types: weather, remote-sensed vegetation, land cover, habitat and mast. Weather and vegetation features contain a value aggregated in a particular time window. Land cover, habitat and mast features contain the value of land cover in a point, the type of tick habitat in the sampling sites, and the strength of a mast year for three tree species, respectively. The remaining of this section describes how the features associated to each type were obtained from the original data sources.

**Table 1:** List of features involved in the current analysis

ID	Feature name	Short description	Type
1	Litter	Thickness of litter layer (Gassner et al. [15])	H
2	Moss	Coverage on a 1–10 scale of the moss layer	H
3	Herb	Coverage on a 1–10 scale of the herb layer	H
4	Brush	Coverage on a 1–10 scale of the brush layer	H
5	Tree	Coverage on a 1–10 scale of the tree layer	H
6	BioLC	Land cover as described in [15]	H
7	Oak-Y	Strength of mast year in a oak forests	M
8	AOak-Y	Strength of mast year in American oak forests	M
9	Beech-Y	Strength of mast year in European beech forests	M
16	tmin-X	Average minimum temperature in a time window	W
17	tmax-X	Average maximum temperature in a time window	W
18	prec-X	Average precipitation in a time window	W
19	rv-X	Average evapotranspiration in a time window	W
20	rh-X	Average relative humidity in a time window	W
21	sd-X	Average saturation deficit in a time window	W
22	vp-X	Average vapour pressure in a time window	W
93	min_ndvi	Minimum NDVI value for a particular location in a year	V
94	range_ndvi	Range for NDVI value for a particular location in a year	V
95	min_evi	Minimum EVI value for a particular location in a year	V
96	range_evi	Range for EVI value for a particular location in a year	V
97	min_ndwi	Minimum NDWI value for a particular location in a year	V
98	range_ndvi	Range for NDWI value for a particular location in a year	V
99	lc25 m	Land cover type in a particular location at 25 m spatial resolution	L
100	lc500 m	Land cover type in a particular location at 500 m spatial resolution	L
101	lc1 km	Land cover type in a particular location at 1 km spatial resolution	L

The features belong to the following categories: tick habitat (H), mast years (M), weather (W), vegetation (V) and land cover (L). The weather features are calculated at 11 temporal aggregations, so there are 77 weather features in total. The X character is replaced by a number between 1 and 7 for short-term temporal aggregations or by a 14, 30, 90 or 365 in the case of bi-weekly, monthly, seasonal or yearly temporal aggregation, respectively. Mast features have a Y character that will be replaced by a number between 0 and 2 in function of the mast year they are referring to. In total, there are 101 features involved in this work

Because of the lack of consensus in the literature on the optimal temporal unit(s) to model AQT, we created a suite of features by aggregating each weather variable (i.e. minimum and maximum temperature, precipitation, evapotranspiration, relative humidity, saturation deficit, and vapour pressure deficit) at multiple temporal scales. These temporal scales are defined by the number of days before the date of the tick sampling. The reason for doing this is straightforward: we assume the tick count produced today, depends on past weather conditions. Therefore, for each tick sampling date we calculated weather features using a range of 1–7 days before the sampling date (i.e. fine temporal units), and of 14, 30, 90 and 365 days (i.e. coarse temporal units). This procedure leads to 11 features per weather variable, adding up a total of 77 features (indices 16–92, type W).

Using GEE, we averaged the 3–4 images available per month to reduce the impact of clouds. Then, using the coordinates of each of the flagging sites, we obtained three (NDVI, EVI and NDWI) time-series summarizing the evolution of vegetation indices since 2005. To remove further noise in these time series, we decomposed each of them into their seasonal, trend and noise components. We kept the seasonal component and obtained the minimum value and range (i.e. width between the minimum and maximum values) per transect and vegetation index. This procedure creates 6 vegetation features (indices 93–98, type V) that condense the general vegetation and moisture conditions in the site over the time-series.

For the land cover, we reduced the number of classes to 12 due to two reasons: (1) the flagging sites are located only in certain types of land cover (e.g. deciduous, grasslands); (2) several land cover types are unrelated to the tick ecology (e.g. sweet water, saltmarshes) or can be aggregated to a coarser level (e.g. types of crop to agricultural land), thus can be unified in a single category. After re-classifying the LGN, the product was resampled to 500 and 1000 m of spatial resolution using a majority filter. This process allows to account for the surroundings of each flagging site, and reduces the chances of the flagging site to be placed in a noisy pixel at 25 m resolution. We obtained the value of the land cover for each of the flagging sites at these three different spatial resolutions (indices 99-101, type L). The strength of the mast year of the year of the observation, as well as the strength of the previous 2 years (indices 7–15, Type M) is included in our work, because tick dynamics might have a delayed response to mast years. Finally, the habitat characteristics per transect are described using 5 variables: the thickness of litter layer and the amount of moss, herbal, brush and tree layers, which are encoded in 5 features (indices 1–6, Type H).

## Modelling AQT with Random Forest

Random forest (RF) [6] is an ensemble learning method that can be used both for classification and regression problems. Ensemble methods rely on the creation of a committee of experts, which work on solving a real-world problem while minimize the chances of taking a poor decision. In the case of RF, the ensemble is formed by a group of weak learners called decision trees, which are combined to create a robust decision ensemble.

RF is a combination of the bagging growing scheme [7] and the random subspace method [20]. These two sources of randomness contribute to create an ensemble with very different trees that lead to high variance predictions when tested individually [28]. Bagging allows RF to see multiple variations of the input data, whereas the RSM introduces randomness in the samples and features presented to each tree during the learning phase. This process creates an ensemble of trees, which is capable of adapting to the tick dynamics phenomenon, and yield predictions with great robustness and stability [43].

The mechanism used by RF to grow decision trees for regression problems, such as modelling AQT as a function of environmental features, is conceptually simple. For each tree (*B*), *N* bootstrap samples (with replacement) are drawn from the available training data. This subsample is used to grow a unique decision tree (*T*_*b*_) by recursively partitioning the N samples until a stop condition is reached, namely: (1) all the samples within a node have the same target response target; (2) the samples in the node are homogeneous with respect to the selected features; (3) a heuristic, such as the maximum depth of the tree, is reached. If none of these conditions are met, the algorithm grows the tree by selecting the best feature and split point among a given and random subset of training features, where best means that it minimizes the Mean Squared Error (MSE). This process creates two child nodes, and the available samples are assigned to them considering the split criteria (e.g. samples with values for feature *m* larger than the split point value go to the left child node). This procedure is repeated until a full forest with *B* trees is grown.

After completing the training phase, RF predicts unseen samples by averaging the predictions of the *B* trees. This reduce the variance and the generalization error of the predictions. In fact, the generalization error converges as the number of trees increases, thus reducing the chances of overfitting data [6, 43].

A key characteristic of RF is that it provides a measure of the importance of the features involved in the modelling. This is done by averaging the reduction in MSE associated to the use of each variable in each of the nodes/trees that form the ensemble [28]. In our work, we exploit this characteristic to understand the main drivers of tick dynamics. Thus, the ranking of features provided by RF gives an idea about what the most relevant (or irrelevant) features are, regardless of the dimensionality of the problem. This is particularly suitable to understand the complex and non-linear interactions found in biological and environmental systems.

RF, like most data-driven regression methods, are not time-aware models. This means that its standard application to regression problems involving (seasonal) time-series, such as the AQT dataset, can lead to sub-optimal results. The reason for this is that the trees in the RF ensemble are trained with random subsets of the training set, where each data sample belongs to a particular date. Thus, RF is trained to predict single snapshots and remains unaware of the temporal continuity of the time-series.

In this work we overcome this limitation by introducing time-awareness in RF. To do so, we transformed the AQT counts into monthly Z-scores by: (1) grouping the 9 years of observations according to the month when they were collected; (2) calculating monthly means and standard deviations, after removing extreme observations from each group so that the Z-scores are not biased. In this context, extreme observations are those that report AQT counts above the 3rd quartile or below the 1st quartile of the monthly values; (3) creating monthly Z-scores, by subtracting the monthly mean from each observation, and dividing the result by the corresponding monthly standard deviation. In this way, we ensure that samples collected during the same month have a constrained and normalized range of AQT counts.

With this monthly normalization we train RF to understand which factors increase or decrease AQT with respect to the long-term average, instead of modelling the absolute number of ticks recorded in a particular location and month. Moreover, by predicting monthly Z-scores we help RF to understand the temporality of the data and hope to get more realistic seasonal dynamics than by using the classical (single snapshot) RF model.

The general set-up of the RF models was as follows: (1) we reserved 70% of the data for training, and the remaining 30% was used for testing the model. Samples were randomly assigned to the training and test subsets; (2) To account for the randomness of RF (different features and samples used in each tree/run), we executed the models 10 times (keeping the training and test samples constant) and the error metrics and feature importance were averaged; (3) we use two well-known statistical metrics to validate our results, the root mean squared error (RMSE) and the normalized RMSE (NRMSE). Note that the error metrics were obtained after de-normalizing the Z-scored signal. Finally, we used the trained model to prepare maps illustrating its performance in a country-wide scenario.

## Experiments

The process described in “Volunteered tick counts reports” section creates four versions of the original dataset with a varying number of zero and peaky AQT, and the process of feature engineering from “Characterizing the environment” section enriches each of the volunteered observations with 101 features. With this set-up, we designed the tree experiments explained in the next sub sections,whose goal is: (1) to assess the impact of noisy observations and selecting the best model capable of capturing AQT dynamics; (2) to evaluate the most important features to model AQT at different time scales; (3) to create AQT map for forested areas in the Netherlands.

### Model selection by assessing the impact of noisy AQT

**Fig. 2:**
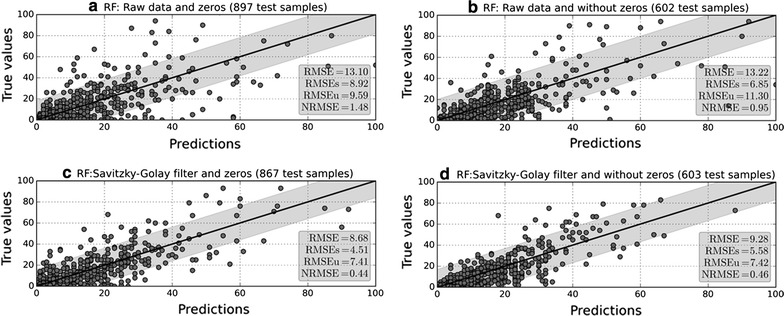
Performance of RF in each of the four selected scenarios. The X-axis represents the predictions yielded by the model and the Y-axis the true values measured by volunteers. To assess the quality of the volunteered AQT dataset, we have tested RF with varying levels of zero AQT and peaky AQT. As seen, the model has more difficulties in capturing peaks than zeros. Thus, the selected model for the following experiments is the model at the bottom left, because it presents the lowest metrics

We modelled the four versions of the volunteered AQT dataset with our time-aware version of RF. Figure 2 shows the general performance of the models. To ease the interpretation of these results, three elements are included: (1) a 1:1 line showing the ideal predictions; (2) a grey band showing one standard deviation from the mean of the observations; (3) a grey box containing the selected statistical metrics for this experiment. The visual inspection of the four plots shows that the two experiments using raw data perform poorly when compared to the two experiments using smoothed data. The models built with raw data have the highest errors in terms of RMSE and NRMSE and also present a higher dispersion of the predictions, indicating that these models did not properly capture the peaky AQT observations.

A close inspection of the NRMSE metric reveals that RF models with smoothed data present very similar performances, regardless of the number of zeros left in the AQT dataset. This suggests that smoothed models can capture the conditions yielding low AQT, but peaky AQT may be actually hampering the modelling process. This is clearly visible when inspecting the points falling outside the gray band in the bottom subplots: a certain number of high AQT true observations could not be captured by the model, thus producing a lower prediction than the true value. We selected the model for next experiments based on the lowest RMSE and NRMSE metrics, thus, out of the four models, we picked the one keeping zero AQT and smoothing the peaky AQT with the Savitzky-Golay filter.

Table 2 presents the feature importance of the top 10 features for the selected RF model. To ease the interpretation of results, we restrict the ranking of the feature importance to the top 10 most prominent out of 101. As seen in this table, the modelled phenomenon is driven by a combination of several weather variables and a vegetation one. The two most explanatory features are the annual evapotranspiration (i.e. ev-365) and the monthly relative humidity (i.e. rh-30). Temperature, which has been traditionally spotted in tick modelling studies as a major driver of tick dynamics, only appears once (as tmax-365) and with a relatively low importance. In this experiment, water-related features perform better than temperature. Note that evapotranspiration and relative humidity do appear several times in the ranking (i.e. ev-90, rh-365), suggesting that in a context with multiple atmospheric variables, water-levels are again more important than temperature. It is also important to highlight that variables about mast years or tick habitat do not appear in the top ten. This could be because they are static (i.e. one value for the whole study period) and, hence, unable to explain the temporal and spatial variation in seen in the AQT dataset.

**Table 2:** Ranking of the top ten most important features (out of 101) for the selected RF model

Position	Feature	Importance
1	ev-365	15
2	rh-30	11
3	tmax-365	7
4	prec-90	4
5	prec-3	4
6	ev-90	3
7	rh-365	3
8	tmin-365	2
9	prec-365	2
10	tmax-90	2

The sum of the feature importance for all features provided by RF equals to 1, but to ease the interpretation of results we multiplied it by a hundred to have natural numbers. As seen, features involving atmospheric water levels (i.e. evapotranspiration and relative humidity) are found to be important to predict tick activity, since they appear several times in the current ranking

**Fig. 3:**
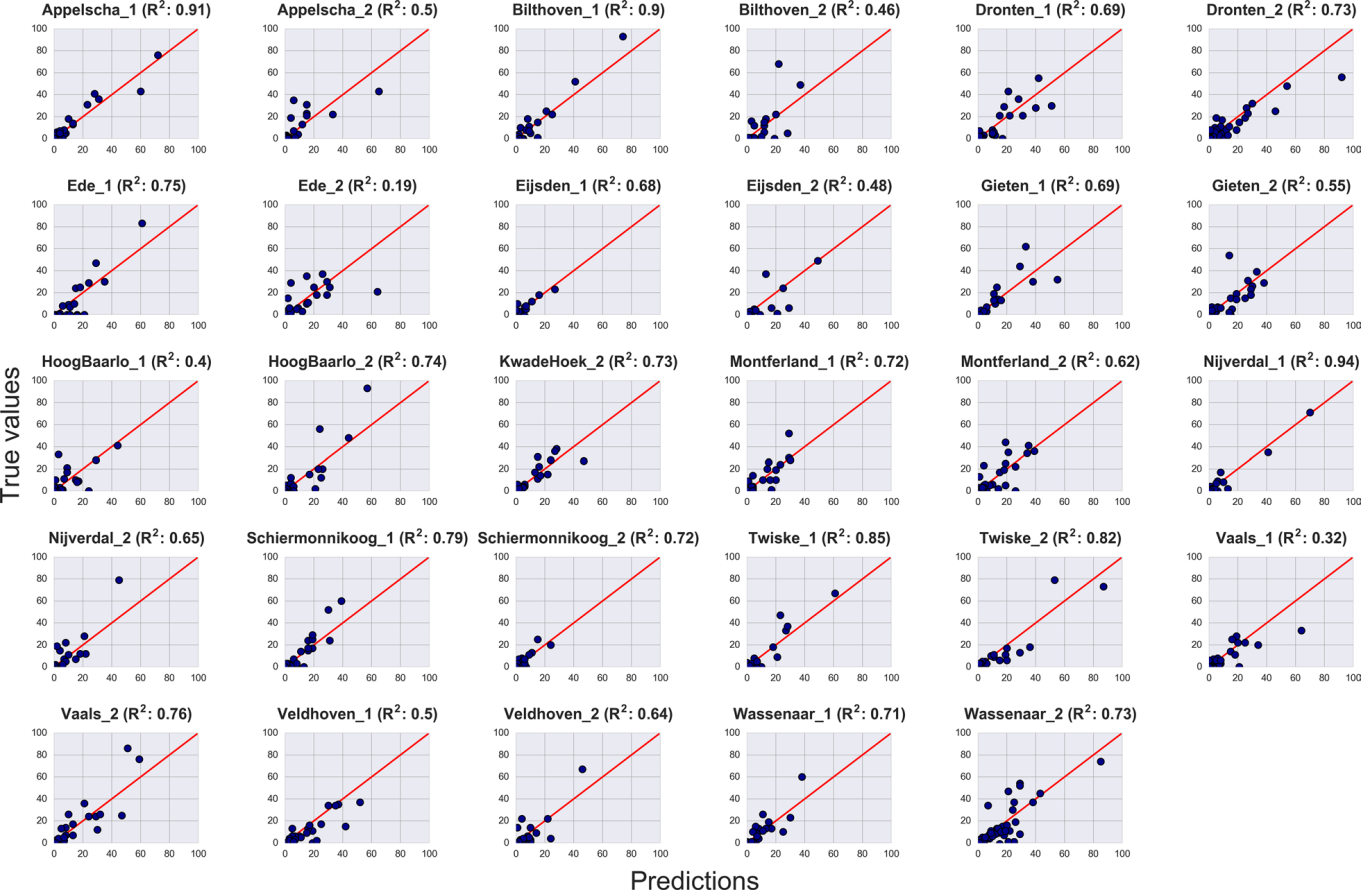
Performance of the selected RF for each flagging site. The X-axis represents the prediction yielded by the model and the Y-axis the true values measured by the volunteers. The R^2^ value is provided in the title of each subplot

**Fig. 4:**
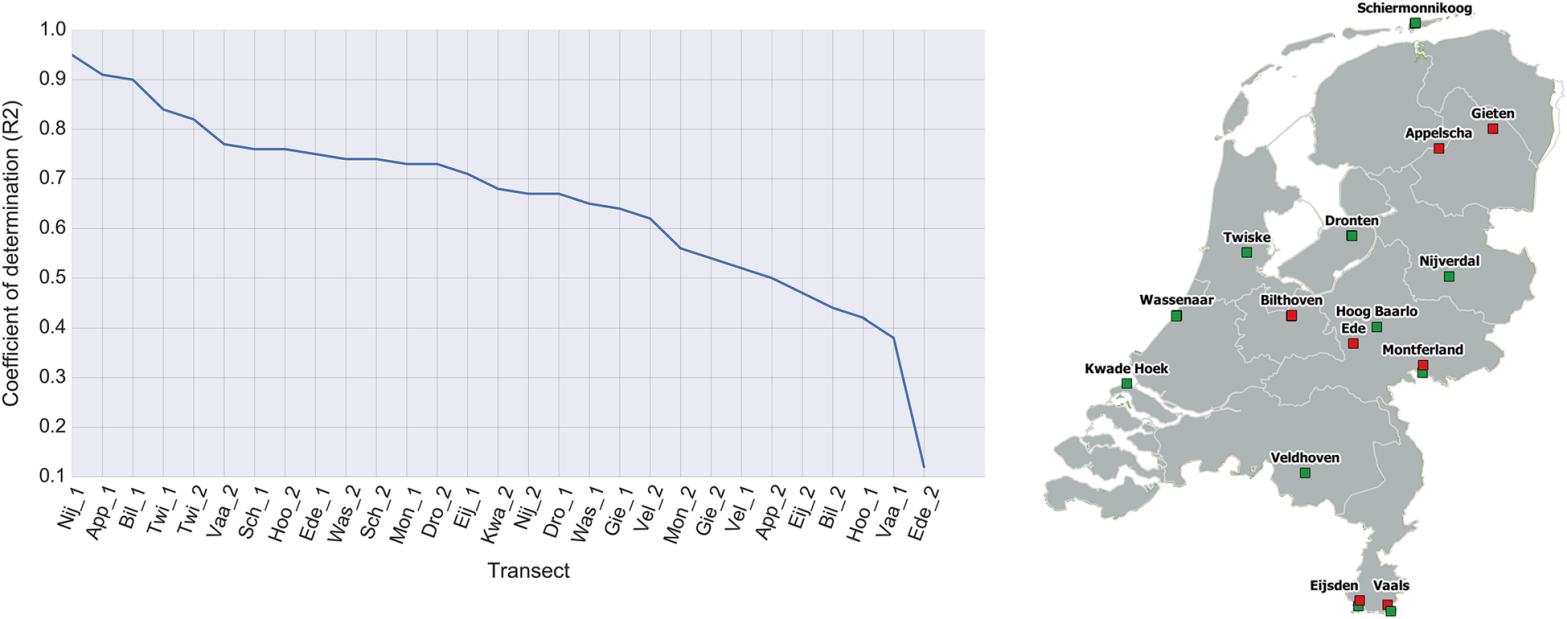
Performance of the selected RF model per transect sorted by the R^2^ score (left) with its geographic location (right). The left image shows that the model is able to fit half of the sites with a moderate-to-strong R^2^ (i.e. 0.7 < R^2^ < 1), whereas the performance in the remaining sites is weak-to-moderate (i.e. 0.3 < R^2^ < 0.7). This means that the transects with a high R^2^ score, respond better to weather variables than the rest of the transects, which may be driven by variables not included in the current model (e.g. wildlife) due to inavailability. The right figure shows the geographic location of the transects: green squares represent transects with moderate-to-strong fitting, whereas red squares represents transects with weak-to-moderate fitting

To further evaluate the usefulness of our RF-based model to predict AQT, we split the test samples according to their associated transect (cf. Fig. 3). The goodness of the fitting (R^2^) between the predictions and the real smoothed AQT values varies between 0.19 and 0.94, indicating that the performance of the model strongly depends on each transect. In Fig. 4 (left) we sort the R^2^ values to provide a better depiction of the performance of the model per transect. Based on these results, we note that the model presents a moderate-to-strong R^2^ (i.e. 0.7 < R^2^ < 1) for roughtly half of the sites. This means that these transects better respond to weather variables than the remaining transects, in which AQT may be driven by variables, such as wildlife, not included in the current model. Note that for transects within the same site (e.g. Vaals, Montferland) the goodness of fit is very different, revealing the very local nature of AQT. Figure 4 (right) shows the geographic representation of the transects. Symbols in green represent the transects better responding to weather variables, whereas red symbols represent the poorly fitted transects. The visual inspection of this figure shows no strong spatial pattern (e.g. north–south gradient).

### Feature importance across multiple time scales

The model structure selected in the previous section is used here to find out the best temporal scale to model AQT. To do so, we train one RF model for each of the 11 time scales described in “Characterizing the environment” section and we execute the model with a subset of features of the input dataset: we keep all the non-weather features (a total of 24 features) and we add the weather features corresponding to that particular time scale (7 features). Thus, we run the modelling process 11 times with 31 features, providing at each iteration the feature importance. In this way, it is possible to get new insights about whether the importance of the features to model AQT change over increasing temporal windows, which might guide the choice of a particular time scale to model AQT optimally.

Table 3 shows the importance of the features at multiple time scales. Each column of the table shows the top five most important features (out of 31) for each of the selected time scales. To ease the description of results at multiple time scales, we restrict the ranking of features to the most relevant top five. This table shows that the most explanatory features for all time scales are weather-based ones and that non-weather features (i.e. vegetation, land cover, tick habitat, mast years) do not significantly contribute to model tick dynamics. Evapotranspiration, relative humidity and the maximum temperature appear to be the most important features. EV better performed in the short-term experiments (i.e. temporal aggregation from 1 day to 4 days before the sampling date), whereas RH is the best one in the long-term experiments (i.e. seasonal and annual temporal aggregation). TX appears to be the best predictor in the remaining experiments.

**Table 3:** Ranking of the top five features for the selected RF model across all temporal scales

	Days																					
	1		2		3		4		5		6		7		14		30		90		365	
	F	I	F	I	F	I	F	I	F	I	F	I	F	I	F	I	F	I	F	I	F	I
Ranking																						
1	EV	15	EV	15	EV	15	EV	15	TX	16	TX	14	EV	14	TX	14	TX	15	RH	17	RH	16
2	TX	13	TX	14	TX	14	TX	14	EV	13	EV	13	TX	13	TN	13	PR	14	EV	15	TX	15
3	TN	11	TN	12	PR	13	PR	11	TN	12	P	13	TN	12	EV	12	RH	12	PR	14	PR	13
4	RH	11	RH	11	TN	12	TN	11	PR	11	RH	12	RH	12	PR	12	TN	12	SD	9	EV	13
5	PR	10	PR	9	RH	10	RH	10	RH	10	TN	9	PR	11	RH	11	EV	11	TN	9	TN	12

Each feature is accompanied by its importance, which has been calculated as the mean of 10 runs. Features involving atmospheric water levels (i.e. evapotranspiration and relative humidity) are found to be relevant to model tick activity in all temporal scales. These results are consistent with the ones provided by the general model. Interestingly, evapotranspiration is marked as the most relevant feature in very short-term time scales, whereas relative humidity is a better predictor for long time scales

### Mapping tick dynamics

The RF model selected in “Model selection by assessing the impact of noisy AQT” section was used in a country-wide exercise to produce three map products: the mean and standard deviation of AQT for the year 2014, and the AQT on a date expected to be close to the peak activity of ticks in nymphal stage. We selected the year 2014 because it is the last of the AQT time-series.

Since the flagging sites are located in forested areas, we identified forested pixels in the land cover map and extracted their locations. Then, to this selection of pixels, we applied the process of feature engineering described in “Characterizing the environment” section for each day of the selected year, thus obtaining 365 country level datasets. The model was retrained with the 86 features available at the country level (i.e. remove tick habitat and mast year features) and tested with the newly created datasets for forested pixels. The predictions yielded by the model were transformed into raster format to obtain three products: first, we obtained the annual mean and standard deviation of AQT based on the daily computed values, which identifies at the country level regions with higher or lower tick activity; second, we obtained the temporal profile of the pixels containing the sites to visualize the daily seasonality of AQT; third, we mapped the AQT for a particular day of the year, which is expected to be close to the peak of tick populations in the nymphal stage.

**Fig. 5:**
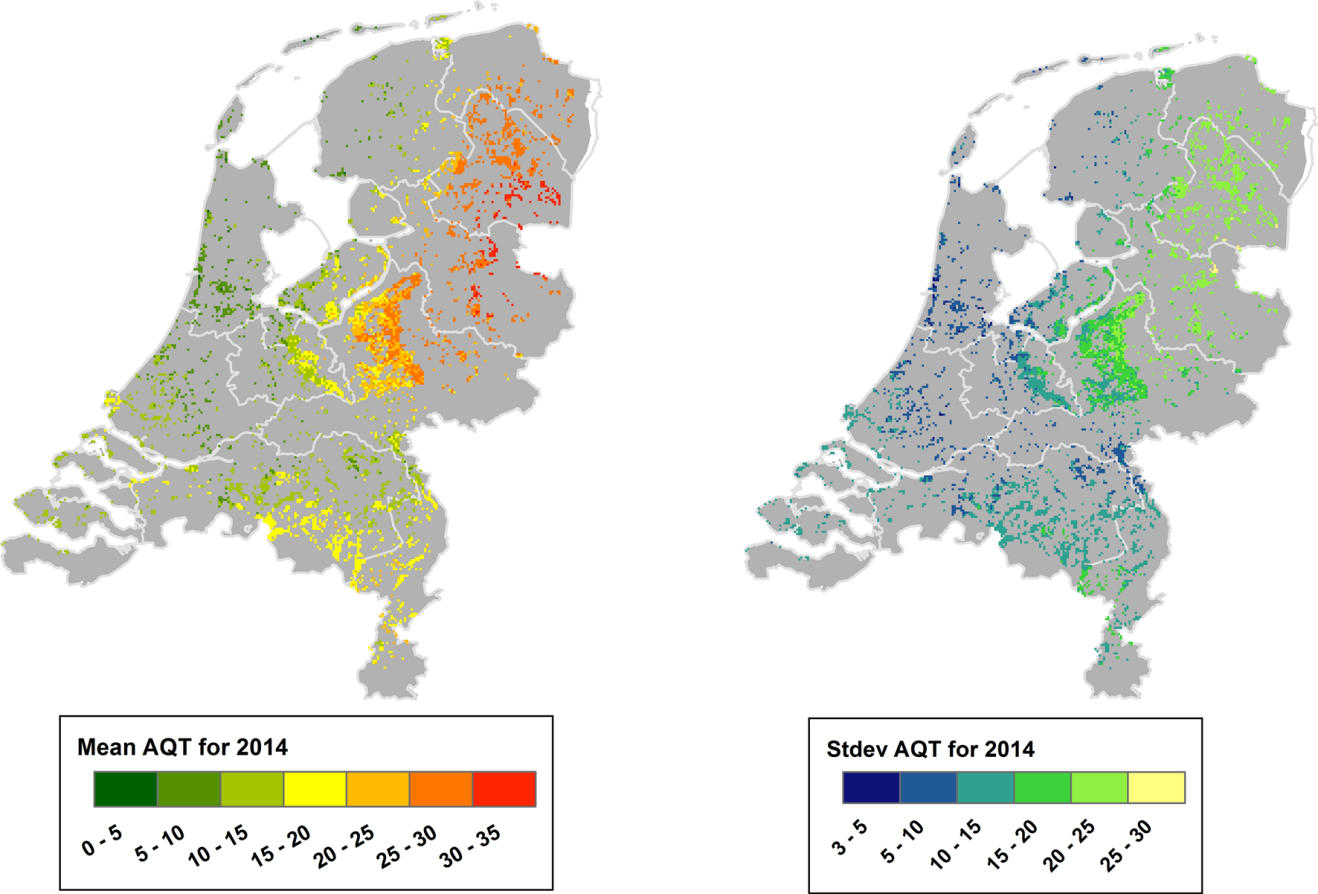
Predicted mean (left map) and standard deviation (right map) of AQT for 2014. The map of mean AQT ranges between 0 (green) and 32 (red) and shows how the highest values of tick activity during 2014 are concentrated within the provinces of Overijssel and Drenthe. The map of the standard deviation of AQT ranges between 3 (dark blue) and 26 (yellow) and shows the deviation from the mean of all forested pixels in the country. As seen, the east half of the country presents higher variations, indicating that tick activity in this areas may change significantly. On the contrary, the predictions along the coastal provinces (i.e. Noord-Holland, Zuid-Holland and Zeeland) do not seem to deviate significantly from the predicted mean

**Fig. 6:**
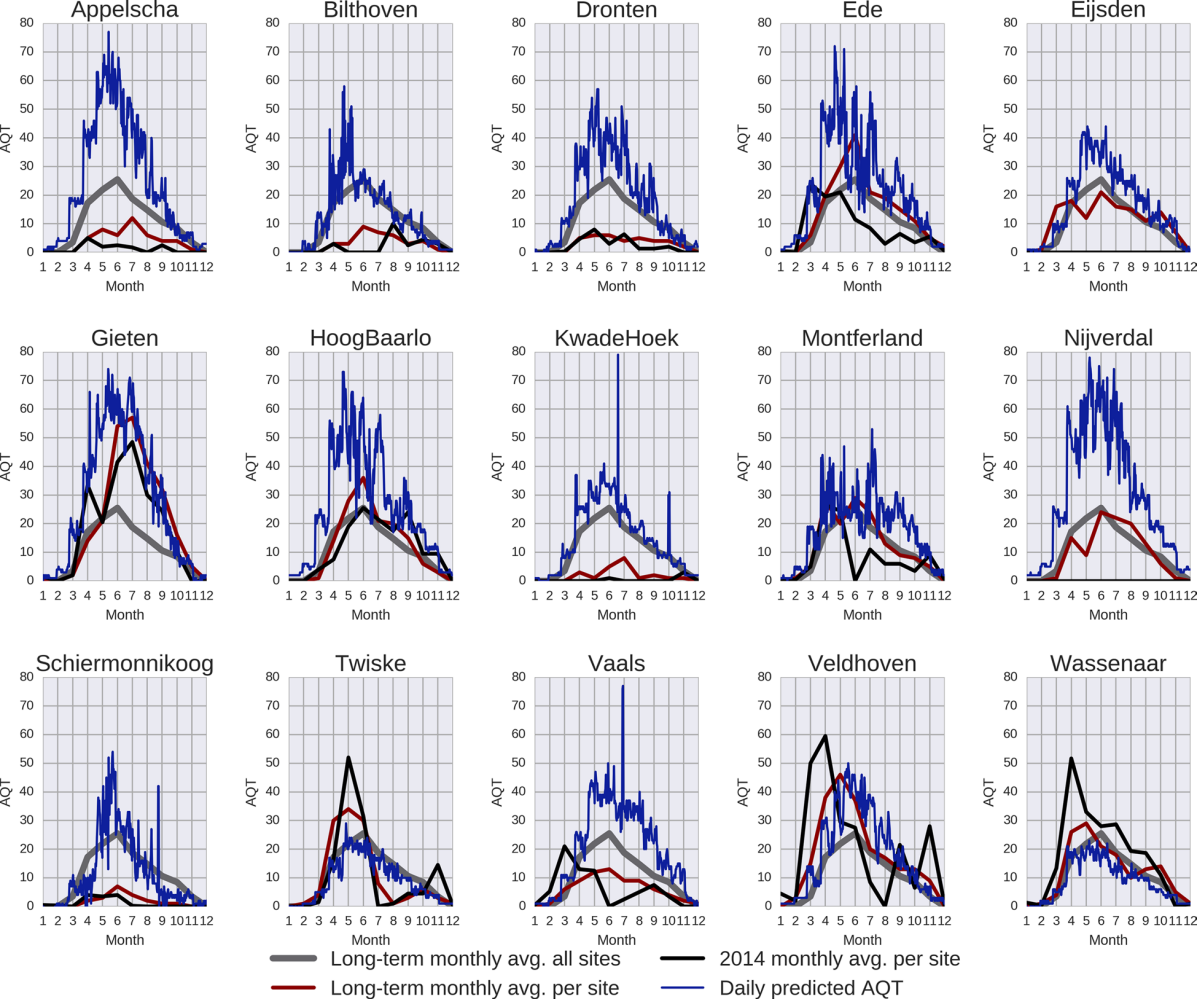
Daily predicted AQT for the locations of the flagging sites. Each subplot contains four curves: the long-term monthly average (grey) across all sites, the long-term monthly average for the flagging site (dark red), the 2014 monthly average for the flagging site (black), and the daily predicted AQT for 2014 yielded by RF (blue). Note that in 2014, the sampling stopped in Nijverdal and Eijsden

**Fig. 7:**
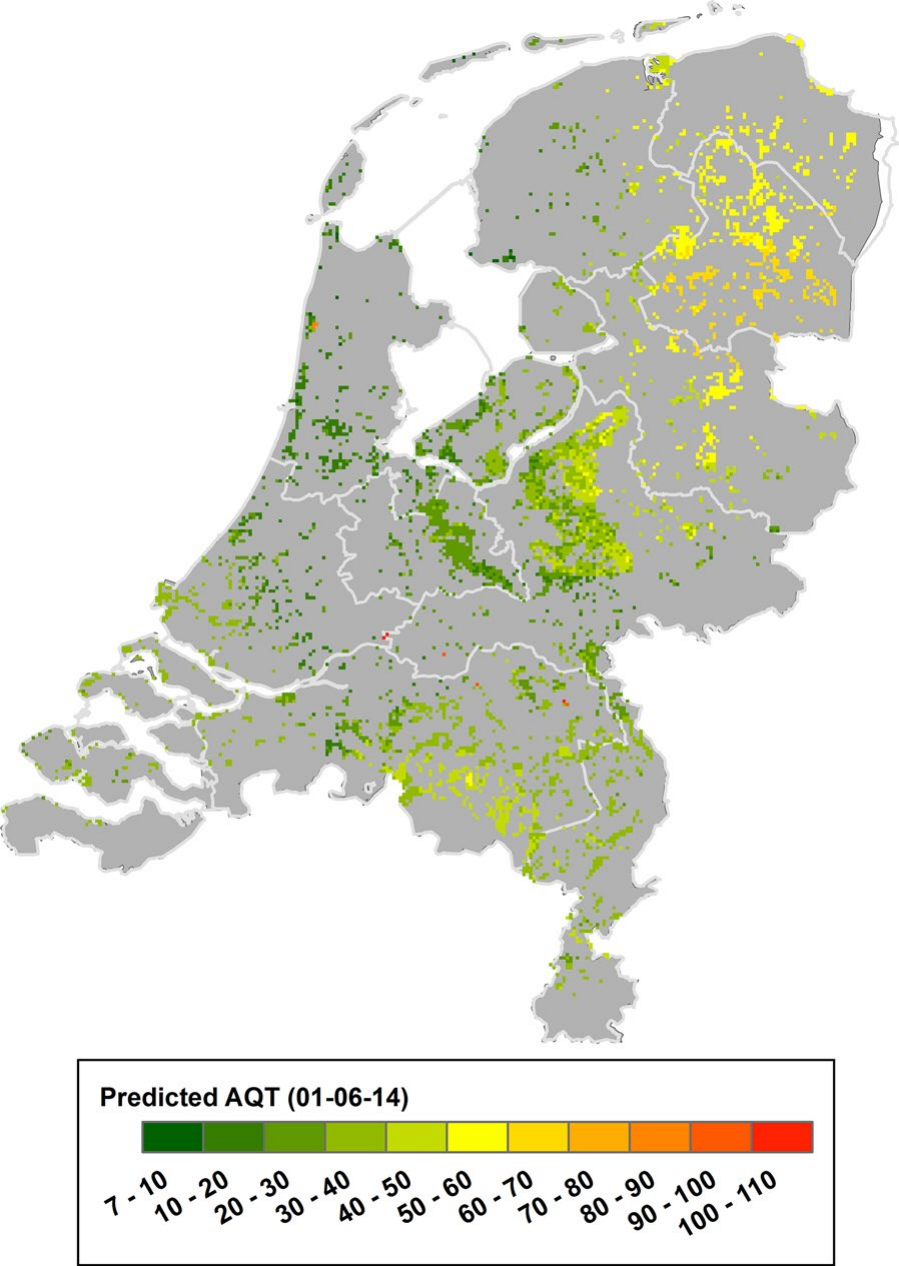
Predicted AQT at the country level for June 1st of 2014. The map ranges from low values of tick activity (dark green) to high values of tick activity (red). The peak populations of ticks in the nymphal stage reaches its maximum between May and June depending on the weather conditions of the year. Thus, we expect this date to be a close depiction of the tick activity at its maximum. The highest AQT are predicted in the east half of the country, in particular within the province of Drenthe, whereas coastal regions present lower levels of tick activity

Figure 5 illustrates the annual mean (left) and standard deviation (right) of predicted AQT. A visual inspection of the mean map shows that there are more AQT in the eastern half of the country (i.e. orange to red regions), especially within the provinces of Overijssel and Drenthe. The standard deviation map depicts the spatial variability of the predictions: regions in light green and yellow show areas where the predictions oscillated significantly above or below the mean AQT, whereas regions in dark blue show locations where the prediction is stable throughout the year. Figure 6 shows the daily temporal evolution of AQT for the grid cells where the flagging sites are located. This figure also shows three additional elements: the long-term monthly average for all sites obtained from the boxplots, the long-term monthly average for the site, and the 2014 monthly average for the site. Note that there are 15 sub plots, because each grid cell overlaps the two transects. This allows to visually identify sites whose predicted AQT is (dis)similar to the averages. Figure 7 shows the predicted AQT for June 1st, which we expect to be close to this peak population of nymphs. As seen, the highest predictions of AQT are predicted in the east half of the country, but there is another spot of high activity in the southern province of Noord-Brabant.

## Discussion

Ensemble learning algorithms such as RF can model non-linear relationships in complex natural processes. This data-driven algorithm provides an indication of the relative feature importance of the predictors involved in the analysis. This is particularly useful to model processes in which the main drivers are unknown. RF has a robust and stable behavior when handling potentially noisy data, a condition often occurring in volunteered datasets, like our AQT time-series. However, since RF is not a time-aware method, it performs sub optimally when modelling seasonal phenomena. In this work, we provide a methodological innovation to introduce time-awareness in RF by transforming our target AQT signal into a monthly bounded one, thus helping the trees in the ensemble to distinguish time.

The study on the importance of the features show that water-related features (i.e. evapotranspiration and relative humidity) are better predictors of the tick activity than temperature or vegetation. This suggests that the tick activity may be driven by atmospheric water levels, which are crucial for tick survival. A closer look of the model performances regarding statistical metrics, indicate that the model can fit the AQT signal for half of the transects, but the fitting decays for the other half. A hypothesis that may explain this difference is that the tick activity may be driven by different variable depending on the geographic location: the sites with a higher R^2^ score might be strongly influenced by atmospheric conditions, whereas the sites with a lower R^2^ score might be driven by variables currently not included in the model (e.g. wildlife). In addition, the analysis of the feature importance at multiple temporal scales is consistent with the results of the general model, because water-related features are spotted as the most prominent features across all temporal scales.

The two major hurdles encountered during this experiment are related with the weather uniformity in the country and the low spatial resolution of the available environmental datasets. First, the low elevation and the small size of the Netherlands make the country very uniform in terms of weather and vegetation variables (e.g. reduced north–south temperature gradient, high and persistent “greenness”). This means that vegetation indices, often included in previous studies in the field, are not discriminative enough to model AQT. Second, the available weather and vegetation datasets have a spatial resolution which is too coarse to model tick dynamics, a phenomenon which was found to be very local as suggested in [14]. This might have an impact when characterizing the AQT with the environmental datasets: different locations with similar weather conditions yield an uncorrelated number of AQT, masking the relationship between weather and ticks and increasing the errors of the model. To mitigate these effects and decrease the average error of the models, we recommend using weather datasets at a finer resolution or involve more volunteers in this long-term citizen science project to get more data.

## Conclusion

Citizen science initiatives allow monitoring of environmental phenomena via crowdsourcing, and produce geospatial data collections that can support scientific analysis. The question at the beginning of this work was whether the collective effort carried out by a group of volunteers, would translate into predictive models estimating tick activity in the Netherlands. Results show that combining volunteered AQT data with environmental variables and modelling them with a time-aware version of RF, can capture most of the spatial and temporal variation in the number of active questing ticks in the country.

The combined analysis of volunteered AQT and environmental variables consistently spotted that water-based features, especially evapotranspiration, play a crucial role in predicting AQT. In this sense, further studies in the field of tick ecology should consider adding, besides the classical temperature and vegetation indices, water-based features. Aside identifying the most important variables to model tick dynamics, this study has produced a model that, scaling up from volunteered observations, can map daily tick activity at the country level. The use of this model may open the way to study spatial patterns and seasonal trends at the national level, not only tick activity, but also of other non-linear natural phenomena, such as phenological events or species distributions.

With these new insights, we envision different applications in the field of tick related ecological research, nature management and public health. In ecological research, our tick activity model allows the identification of tick hotspots and of the sampling sites where the model fit was good or bad, which can be used to better select new monitoring sites. With the model we can better analyze the impact of extreme weather events and climate change on tick dynamics and population development. In nature management, these maps can help owners of green areas to be more aware of the variation of tick dynamics in the areas they are responsible for, which can lead to a better planning in space and time of different forestry management activities. In tick hotspots with many visitors, nature managers could consider to more frequently mow the grass directly next to walking and cycling trails or picnic areas to try to reduce tick populations. In public health, this model can be used to better inform people that visit natural areas on current tick activity levels. In combination with weather forecasts also detailed forecasts for the coming days can be given. The proposed model predicting tick activity will replace the very basic tick activity forecast currently implemented in the Dutch citizen science website Tekenradar (Tick radar, www.tekenradar.nl). The spatially detailed tick activity forecasts are expected to raise awareness among the general public and many different stakeholders involved in the problem of Lyme disease. Having more detailed information hopefully translates in an increase of protective and preventive measures when visiting forested areas. Overall, we expect that a better understanding of tick dynamics may contribute to design interventions to reduce the incidence of Lyme disease.

## Footnotes

1
https://data.knmi.nl/datasets.

2
https://code.earthengine.google.com/.

3
https://earthengine.google.com/.

4
http://tinyurl.com/j47m2ol.
